# Alectorioid Morphologies in Paleogene Lichens: New Evidence and Re-Evaluation of the Fossil *Alectoria succini* Mägdefrau

**DOI:** 10.1371/journal.pone.0129526

**Published:** 2015-06-08

**Authors:** Ulla Kaasalainen, Jochen Heinrichs, Michael Krings, Leena Myllys, Heinrich Grabenhorst, Jouko Rikkinen, Alexander R. Schmidt

**Affiliations:** 1 Department of Geobiology, University of Göttingen, Göttingen, Germany; 2 Department of Biology and Geobio-Center, University of Munich (LMU), Munich, Germany; 3 Department of Earth and Environmental Sciences, University of Munich (LMU), and Bavarian State Collection for Palaeontology and Geology, Munich, Germany; 4 Finnish Museum of Natural History, University of Helsinki, Helsinki, Finland; 5 c/o Amber Study Group, Geological-Palaeontological Institute and Museum, University of Hamburg, Hamburg, Germany; 6 Department of Biosciences, University of Helsinki, Helsinki, Finland; Penn State University, UNITED STATES

## Abstract

One of the most important issues in molecular dating studies concerns the incorporation of reliable fossil taxa into the phylogenies reconstructed from DNA sequence variation in extant taxa. Lichens are symbiotic associations between fungi and algae and/or cyanobacteria. Several lichen fossils have been used as minimum age constraints in recent studies concerning the diversification of the Ascomycota. Recent evolutionary studies of Lecanoromycetes, an almost exclusively lichen-forming class in the Ascomycota, have utilized the Eocene amber inclusion *Alectoria succinic* as a minimum age constraint. However, a re-investigation of the type material revealed that this inclusion in fact represents poorly preserved plant remains, most probably of a root. Consequently, this fossil cannot be used as evidence of the presence of the genus *Alectoria* (Parmeliaceae, Lecanorales) or any other lichens in the Paleogene. However, newly discovered inclusions from Paleogene Baltic and Bitterfeld amber verify that alectorioid morphologies in lichens were in existence by the Paleogene. The new fossils represent either a lineage within the alectorioid group or belong to the genus *Oropogon*.

## Introduction

Lichen symbioses represent stable mutualistic associations in which photoautotrophic algae and/or cyanobacteria provide carbohydrates for heterotrophic fungi. This nutritional strategy has been adopted several times independently in various fungal lineages, and today approximately 20% of all known fungal species are lichenized [1–3]. Despite the prevalence of lichens in certain modern ecosystems, their documented fossil record remains scarce. The oldest fossil indicating a lichen-like symbiosis has been described from approximately 600 Ma old marine phosphorite [4], and the earliest records of internally stratified lichens with cyanobacterial and presumably algal photobionts are from ca 410 Ma old Lower Devonian rocks [5–6].

Amber, formed by the hardened resin of trees, is famous for its ability to capture ancient life and preserves even soft-bodied microorganisms in cellular and ultrastructural fidelity. Several well-preserved lichens have been described from 35–50 Ma old Baltic and 16 Ma old Dominican amber (for age estimation, see [7–8]), and identified as representatives of extant genera [9–12]. These rare lichen fossils have served as minimum age constraints in several recent studies assessing the evolutionary history of the Lecanoromycetes [13–15]. There is always some level of uncertainty when morphologies of fossils are incorporated into phylogenetic trees that are based on DNA sequence variation of extant taxa [16–19], but this inherent problem can often be minimized by a thorough inspection of the fossils and assessment of their characteristics [20–22]. An inclusion in Baltic amber described as *Alectoria succini* by Karl Mägdefrau [23] has recently been used as an age constraint for the genus *Alectoria* [13–14]. However, the fossil that formed the basis for *A*. *succini* has not been revisited since the initial publication.

Here we show that the fossil described as *Alectoria succini* does not possess morphological attributes characterizing alectorioid lichens, but rather represents a degraded plant part, probably a root, and consequently is unfit to be used in calibrating ascomycete phylogenies in geologic time. On the other hand, newly discovered fossils from Baltic and Bitterfeld amber demonstrate that *Alectoria*-like morphologies resembling extant lineages within the alectorioid group (*sensu* Miadlikowska *et al*. [24]) or the genus *Oropogon* were actually present in lichens in Europe during the Paleogene.

## Material and Methods

The piece of Baltic amber containing *Alectoria succini* is ~6.2 x 3.5 x 1.2 cm large. It is part of a historical amber collection assembled by Alexander Scheele that is today housed in the Bavarian State Collection for Palaeontology and Geology at Munich, Germany (specimen accession number SNSB-BSPG 1967 XX 1). The amber piece containing *A*. *succini* also includes a spider, spider web, and composite plant hairs of Fagaceae as syninclusions. The Eocene sediments that yield the majority of Baltic amber in the Kaliningrad area (Russia) are 35–47 million years old, whereas fewer specimens are found in up to 50 million-year-old strata [7,25].

The other amber fossils of *Alectoria*-like lichens included in this study come from Baltic and Bitterfeld amber. The fossil contained in Baltic amber is part of the historic Königsberg Amber Collection housed at the University of Göttingen (collection number GZG.BST.21889 [B 14234]). The piece is ~17 x 10 x 7 mm large and contains several fagaceous trichomes as syninclusions. The fossil in Bitterfeld amber is part of the Geoscientific Collections of the University of Göttingen (collection number GZG.BST.27313). The piece is ~9 x 7 x 2 mm large and contains a fagaceous trichome and scattered dark hyphae and conidial chains of a dematiaceous hyphomycete as syninclusions. The amber piece was collected from the Goitzsche mine near the city of Bitterfeld in central Germany and was recovered from the Chattian 'Bernsteinschluff' Horizon in the upper part of the Cottbus Formation. The Upper Oligocene amber-bearing sediment has an absolute age of 23.8–25.3 million years [26–27].

Baltic amber is usually considered at least 35 million years old [25]; however, providing a precise minimum age for Bitterfeld amber is still challenging. There is conflicting evidence regarding whether the uppermost Oligocene age of the amber-bearing strata of the Bitterfeld amber mine reflects the actual age of the Bitterfeld amber. The fact that there is a significant proportion of identical arthropod morphologies in amber from both localities resulted in the hypothesis that Bitterfeld amber perhaps represents re-deposited Eocene Baltic amber [28]. Re-deposition of Baltic amber is, however, unlikely considering the overall geological setting of the Bitterfeld strata and palaeogeographic conditions [25], but local reworking of pre-Chattian amber in the Bitterfeld area has not been refuted so far (see [29], for discussion). In any case, Bitterfeld amber is also Paleogene in age, and its minimum age should be regarded as 23.8 million years.

**Fig 1:**
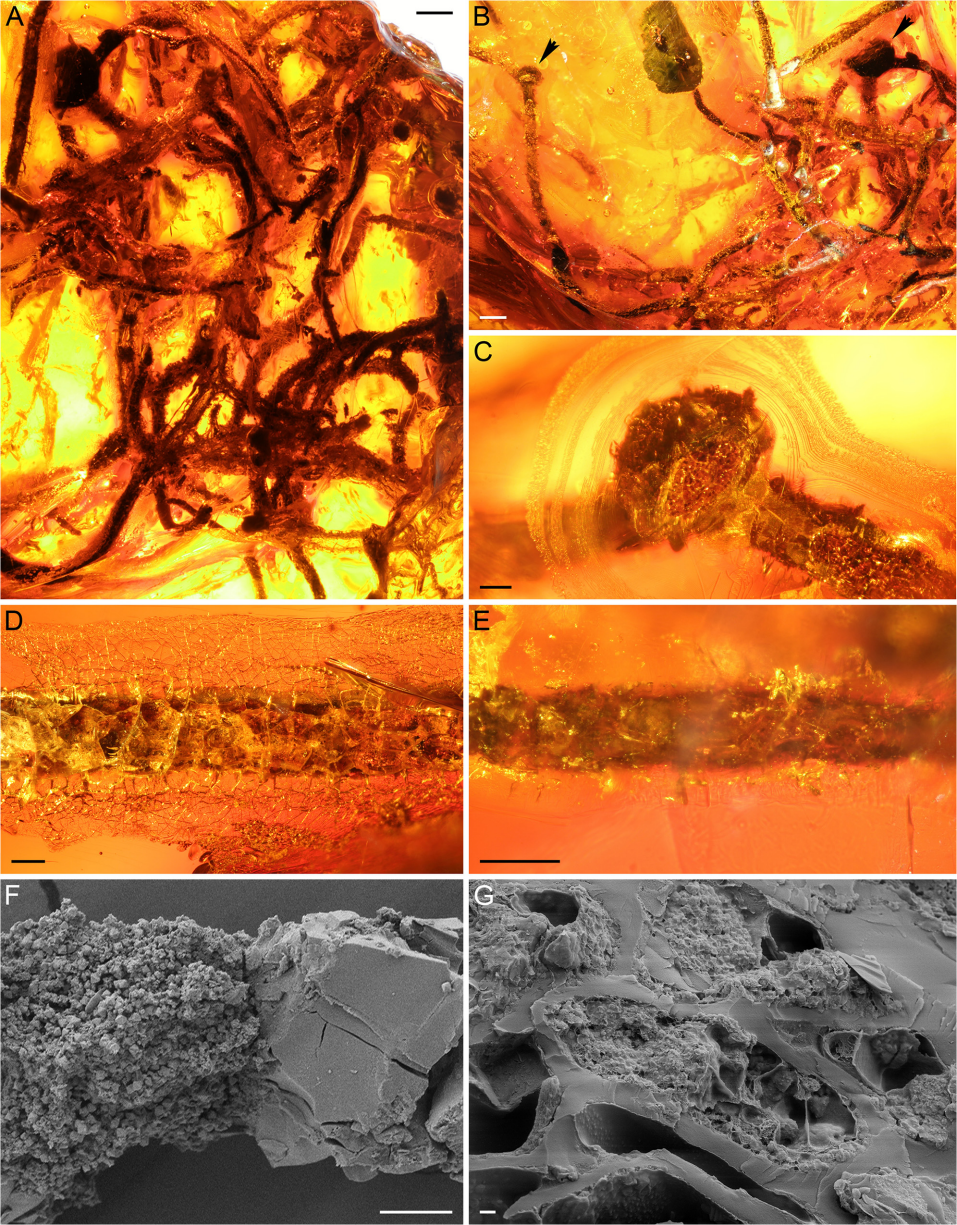
Light (A–E) and scanning electron (F, G) microscope images of *Alectoria succini* (specimen SNSB-BSPG 1967 XX 1). (A) Overview of specimen (scale bar 1 mm). (B) Fossil imaged the same way as in Mägdefrau’s paper [23] (scale bar 1 mm). Arrowheads point to two thickenings initially interpreted as apothecia. (C) Smaller apothecium-like structure (scale bar 200 μm). Note surface fissures in amber. (D) Portion of fossil showing numerous fissures (scale bar 200 μm). (E) Portion of fossil imaged after adding epoxy under vacuum (scale bar 200 μm). (F) Extracted portion of fossil showing decayed tissue and pyrite crystals (left) and surrounding amber (right) (scale bar 10 μm). (G) Several well-preserved structures resembling parenchymatous cells, some filled with tiny pyrite crystals (scale bar 1 μm).

**Fig 2:**
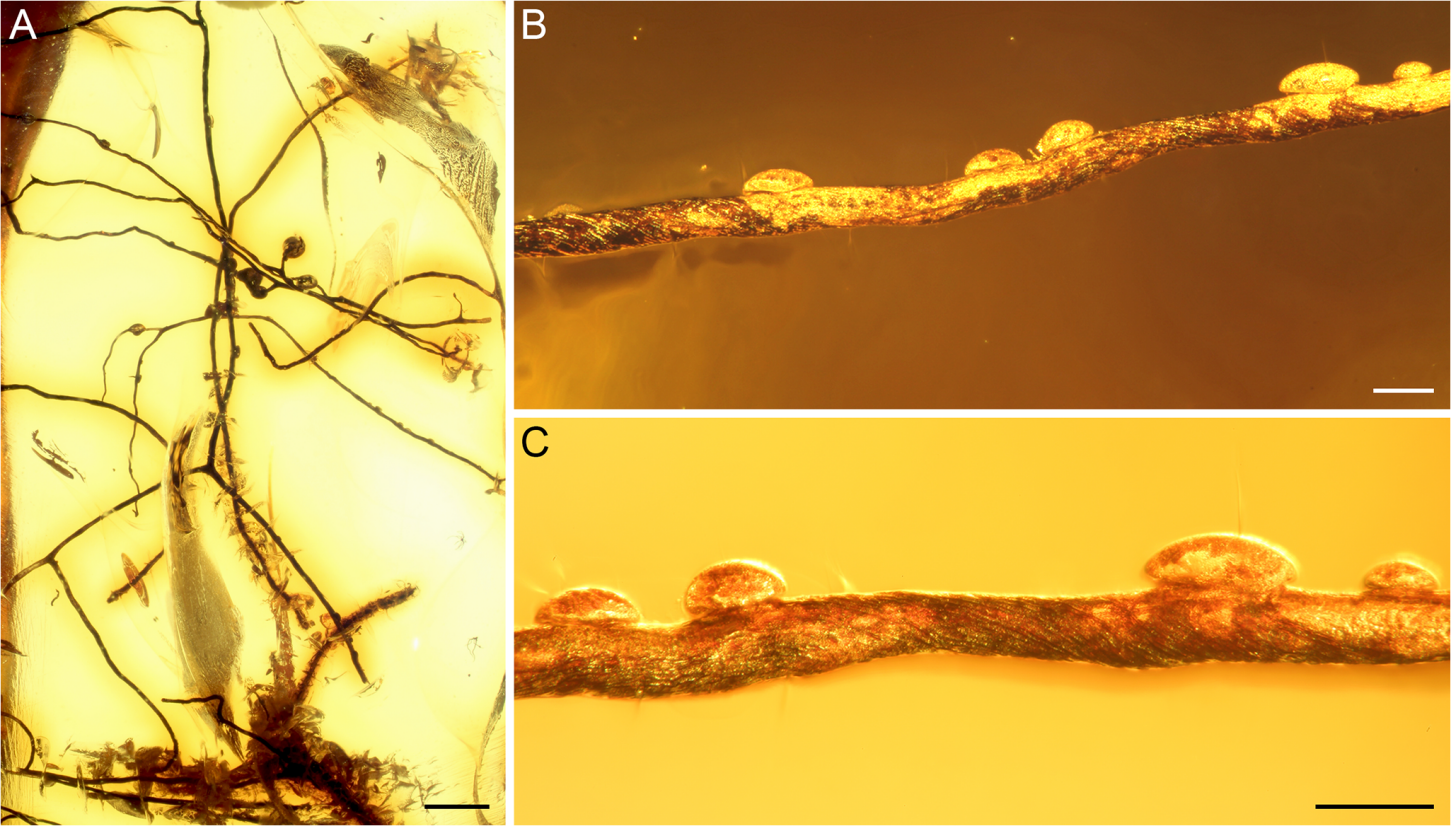
Lichen fossil from Baltic amber (specimen GZG.BST.21889). (A) Overview of specimen (scale bar 1 mm). (B) Branch with possible pseudocyphellae indicated by air bubbles (scale bar 100 μm). C) Detail of specimen, showing twisted branch (scale bar 100 μm).

**Fig 3:**
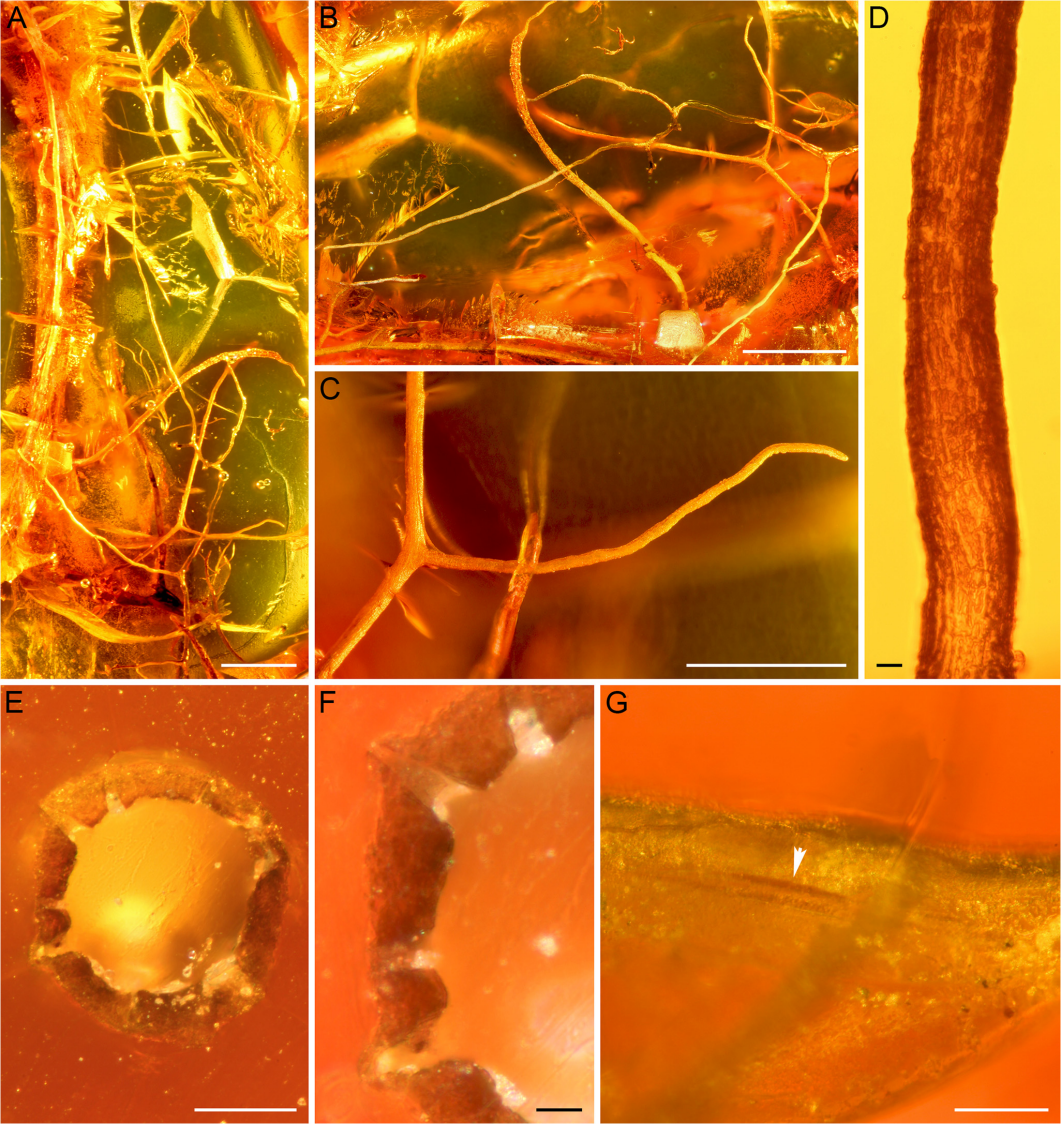
Lichen fossil from Bitterfeld amber (specimen GZG.BST.27313). (A–C) Overviews of specimen (scale bars 1 mm in A and B, and 500 μm in (C). (D) Detail showing surface of one of the thinner branches (scale bar 10 μm). (E) Cross section of one of the thicker branches. Central void or canal in thallus filled with amber (scale bar 50 μm). (F) Close up of perforate formations leading from central void to thallus surface (scale bar 10 μm). (G) Close up of putative linear pseudocyphella on surface of smaller branch (arrowhead) (scale bar 50 μm).

Amber inclusions were studied using a compound microscope (Carl Zeiss AxioScope A1) equipped with a Canon 60D digital camera. In some instances, incident and transmitted light were used simultaneously. Before imaging the new amber pieces were ground and polished manually using a series of wet silicon carbide papers (grit from FEPA P 600 to 4000, particle size 25.8–5 μm, Struers). In order to better illustrate the three-dimensional inclusions, a series of photomicrographs of different focal planes were combined using the software package HeliconFocus 4.45. The images in Figs 1–3 were obtained from the merger of up to 60 optical sections.

For scanning electron microscopy (SEM) minute pieces were removed from a branch of *Alectoria succini*, fixed onto an SEM-mount, sputter-coated with a 12 nm thick Pt/Pd coating using an Automatic Sputter Coater (Canemco Inc.), and examined and imaged with a field emission scanning electron microscope Carl Zeiss LEO 1530 Gemini.

After initial imaging and sampling of the *Alectoria succini* fossil, a drop of a high-grade epoxy (Buehler Epoxicure) resin was applied to one of the filaments reaching to the surface of the amber piece (for protocols, see [30]). When placed under vacuum, the epoxy resin entered the inclusion and surrounding fissures for several millimeters providing a better visualization of the specimen surface. The image of Fig 1E was taken after epoxy treatment.

No permits were required for the described study, which complied with all relevant regulations.

## Results

### *Alectoria succini* Mägdefrau

The inclusion is approximately 20 x 15 mm large (Fig 1A). The opaque fossil consists of elongate, branched structures; three prominent thickenings that occur on the branches (Fig 1B and 1C) have been interpreted as apothecia by Mägdefrau [23]. The fossil is not well preserved because all structures are surrounded by tiny fissures that hinder a more specific visual inspection (Fig 1D). Even after epoxy treatment, only a small portion of the fissures was covered, and no clear image of the surface could be obtained (Fig 1E). The structures believed to represent apothecia (Fig 1B and 1C) are also entirely covered with fissures that prevent in-depth evaluation of their nature. Moreover, they are situated deep within the amber, rendering it impossible to access them without destroying the specimen.

Scanning electron microscopy imaging of the inclusion revealed a highly degraded tissue with numerous tiny pyrite crystals (Fig 1F). However, at the interface of the amber and inclusion, cell-like structures have remained intact, most likely because the resin compounds were able to better penetrate and preserve the tissue (Fig 1G). The cell-like structures are oval in shape, ~24 μm long and up to 8 μm wide, and some appear to be hollow, while others are filled with pyrite crystals. Based on the shape and size, these structures most probably represent remnants of plant cells. Evidence of fungal hyphae, photobiont cells, or any other structures arguing in favor of lichen affinities of the fossil were not found.

### Lichen fossil from Baltic amber

This amber inclusion consists of several slender, terete branches, the longest of which is more than 11 mm long (Fig 2A). Branches are up to 90 μm wide. Branching pattern is wide and some of the subordinate branches extend more or less perpendicularly. The color of the branches appears to be dark brown, and some show twisting (Fig 2B and 2C). All branches are bounded by what appears to be a prosoplectenchymatous cortex composed of hyphae extending longitudinally and spiraling around the circumference of the branch (Fig 2B and 2C). Several small air bubbles are present in the amber along the branches that might suggest the existence of pseudocyphellae which permitted air to be released from the interior of the thallus during embedding (Fig 2B and 2C). Apothecia or vegetative propagules have not been observed.

### Lichen fossil from Bitterfeld amber

The amber inclusion consists of several slender, terete branches, the largest of which is ~7 mm long (Fig 3A). Branches are up to 140 μm wide; there is no clear differentiation into main and subordinate branches (Fig 3A and 3C). Branching pattern is wide, and some of the branches extend perpendicularly. The color of the branches appears to be brownish (Fig 3D). A cylindrical structure seen in cross-sectional view (Fig 3E) represents the dense cortex, while the looser internal layers (photobiont layer and medulla) of the lichen are not preserved. The cortex bounding the branches is up to 20 μm thick and appears to be prosoplectenchymatous and composed of hyphae mostly extending along the long axis of the branches (Fig 3D). The thallus was probably hollow or possessed only a very loose medulla because the center of the branches is filled with amber (Fig 3E). However, evidence of medullary hyphae is not present. The cross section shows several openings reaching from the hollow center to the thallus surface (Fig 3E and 3F). Two of the openings rise slightly above the generally smooth outer surface of the cortex. A linear pseudocyphella of approximately 180 μm long and 10 μm wide is also visible on the surface of one of the smaller branches (Fig 3G). No apothecia or vegetative propagules are visible.

## Discussion

### Identity of *Alectoria succini*

Our re-investigation of the type (and only) specimen of *Alectoria succini* suggests that this fossil most likely does not represent a lichen, but rather comes from a plant, probably from a root based on the branching pattern and the presence of structures strongly resembling a parenchymatous cortical tissue. Unfortunately, the multiple fissures surrounding the inclusion, along with the heavy degradation of the fossil, make a more conclusive assessment of the biological nature of the inclusion impossible. Such superficial fissures frequently develop in amber inclusions as a result of weathering processes, including oxidation [31].

When initially describing *Alectoria succini*, Mägdefrau [23] was convinced that the fossil represented a lichen, but assignment to the extant genus *Alectoria* was regarded tentative. Moreover, the only illustration included in the initial publication (plate XII, figure 5 in [23]) is an overview at low magnification and of relatively poor quality, suggesting that the surface fissures were present already when Mägdefrau studied the specimen. The most important features used by Mägdefrau to interpret the fossil as a lichen are the three prominent thickenings believed to represent apothecia. However, with the exception of overall size which corresponds to the size of typical *Alectoria* apothecia (e.g. [32]), there are no structural details recognizable in the thickenings that could be used to determine their nature. We therefore conclude that the fossil named *Alectoria succini* is in no way persuasive with regard to systematic affinities, and thus it cannot be used as evidence of the existence of the genus *Alectoria* or any other lichen group in the Paleogene.

### New fossils from Baltic and Bitterfeld amber

Both new fossils from Baltic and Bitterfeld amber are quite similar in overall morphology: thin branches with a wide branching pattern, and hyphae primarily oriented longitudinally along the branches (Figs 2 and 3A–3D). The cross-section of the Bitterfeld specimen shows a relatively wide central void or canal, which might represent a natural feature, but could also have been caused by destruction and/or degradation of the medulla prior to or during embedding (Fig 3E). The branches of the Baltic specimen are arranged in a helix (Fig 2B and 2C). Both specimens seem to possess pseudocyphellae: in the Bitterfeld specimen one elongate opening is visible on the surface of one of the branches (Fig 3G), while the Baltic specimen suggests the presence of pseudocyphellae because small air bubbles occur in abundance on some of the branches (Fig 2B and 2C). The pseudocyphellae of both specimens are inconspicuous and likely narrow, because they are rarely visible on the thallus surface. Overall morphology and growth form, together with the presence of a central void or canal in the radially symmetrical thallus and the presence of pseudocyphellae, suggests affinities to the extant lineage of either alectorioid lichens (*Alectoria*-group *sensu* Miadlikowska *et al*. [24]) or, alternatively, the genus *Oropogon*, both belonging to the family Parmeliaceae (Lecanorales). Based on morphological similarities, *Oropogon* was previously also considered part of the alectorioid group [32], but this relationship is not supported by recent molecular studies [24,33]. In addition to molecular characters, the main distinguishing feature between these morphologically partially very similar genera are the ascospores, which unfortunately are not preserved in either of the fossils.

*Oropogon* is a genus of approximately 40 species occurring in eastern and southern Asia and Central and South America [34]. In addition to ascospores, the genus is characterized by predominately isodichotomous branching and conspicuous, perforate pseudocyphellae, which are, however, not present in all species [34]. The central, amber filled void or canal with perforations seen in cross-sections (Fig 3E and 3F) is reminiscent of what can be observed in some extant *Oropogon* species. However, the number of perforations visible in a single cross-section is high (at least seven) in the fossil, whereas extant species usually show only one, or at best two pseudocyphellae per cross-section. However, it is also possible that some of the less prominent perforations in the fossil are of taphonomic origin (e.g. ruptures due to shrinkage of the thallus during embedding).

The monophyletic lineage of alectorioid lichens presently includes five genera of which three produce fruticose, often pendent and beard-like thalli: *Alectoria*, *Bryoria*, and *Sulcaria* [24,33,35]. *Alectoria* is a genus of seven species presently recognized with Northern Hemisphere or bipolar distribution, while *Bryoria* consists of 30–40 mainly circumboreal species, and *Sulcaria* contains six species restricted to either Asia or North America [35–38]. Typically, *Alectoria* produce raised and elongate fusiform to ovoid pseudocyphellae [39] that remotely resemble the slightly raised cracks observed in the cross section of the fossil thallus from Bitterfeld amber (Fig 3E). On the other hand, several species of *Bryoria* produce relatively inconspicuous, linear pseudocyphellae [40] that are similar to the linear pseudocyphella observed on the branch surface of the Bitterfeld fossil (Fig 3G). In most species of *Sulcaria* pseudocyphellae form conspicuous, long, and spiraling grooves [36,41].

Another character distinguishing the aforementioned extant genera is the thickness of cortex: in members of the genus *Alectoria*, this layer is generally thick (50–80 μm), while it is relatively thinner in other alectorioid genera and in *Oropogon* (30–40 μm) [32]. The cortex of the Bitterfeld lichen only reaches up to 20 μm. In addition, the branches in the Baltic fossil are twisted (Fig 2B and 2C), thus resembling the often twisted appearance of the branches seen in *Sulcaria*. Twisting of the branches also occurs in certain species of *Alectoria* and *Bryoria*, but we are not aware of any report in *Oropogon*. In conclusion, even though the two new lichen fossils are morphologically similar to each other, they are not identical. There is a close resemblance to several extant taxa, but the morphological features of the fossils are not of sufficient clarity to assign them to any extant lineage of lichens with confidence.

### Reliable lichen fossils for the calibration of molecular clocks

Many studies of molecular evolution have demonstrated the importance of constraining molecular clocks with fossil evidence (e.g. [42–48]). However, as shown by Taylor and Berbee [49], great care should be taken when choosing fossils because the use of age constraints has significant effects on divergence time estimates and therefore the systematic placement of the fossils is of prime importance. Evaluation of all lichen inclusions in amber described to date shows four fossils that have confidently been assigned to extant genera, the oldest of which is Eocene in age.

Representatives of the genera *Anzia*, *Calicium*, and *Chaenotheca* have been described from Baltic amber. The fossils all are very well preserved, and thus allow reliable identification [10,12,15]. The fossil *Anzia electra* was first described from Bitterfeld amber [10] and later the genus was also identified from a Baltic amber inclusion [15]. *Anzia*, a member of the family Parmeliaceae (Lecanorales), is characterized by the presence of a distinctive spongiostratum composed of one cell wide hyphae [50]. Spongiostratum is a structure of spongy cushions on the ventral side of the thallus formed by anastomosing hyphae. It is a characteristic of only two extant genera, *Anzia* and *Pannoparmelia*, which can be discriminated based on the construction of the spongiostratum hyphae, i.e. uniseriate in *Anzi*a vs. multiseriate in *Pannoparmelia* [50].

*Calicium* and *Chaenotheca* include lichens with pin-like ascomata. Despite superficial similarity, the genera are not closely related, but rather belong to two families, i.e. the Caliciaceae (Lecanorales) and Coniocybaceae (Coniocybales). The fossil *Calicium* sp. consists of a single detached ascoma and of numerous ellipsoidal 2-celled spores [12], which are characteristic of the extant representatives of *Calicium* and clearly distinguish the genus from most other ‘stubble lichens’. The fossil *Chaenotheca* can also be identified with confidence based on the presence of a well-developed thallus, relatively stout and short-stalked apothecia, well-developed excipulum, and spherical, and relatively large 1-celled spores [12].

In addition to the Paleogene fossils, younger (~16 Ma old) lichen fossils from the Miocene have been described from Dominican amber. One of these, the squamulose *Phyllopsora dominicanus* (Ramalinaceae, Lecanorales), closely resembles the extant *Phyllopsora* species [11]. Two fossils described as representatives of the genus *Parmelia* (Parmeliaceae, Lecanorales), i.e. *P*. *ambra* and *P*. *isidiiveteris* [9], most probably do not belong to *Parmelia* as presently circumscribed, but represent other foliose genera within the diverse lineage of parmelioid lichens.

Re-evaluation of *Alectoria succini* might result in a shift of the previously estimated divergence times within the Lecanoromycetes. This is because Mägdefrau's fossil can currently only be replaced by lichen fossils that are not with confidence assignable at generic level. To some extent, the probable alterations can be inferred from the previous analyses run with and without the fossil [13,14]. These analyses showed that the omission of *Alectoria succini* does not significantly change the estimated divergence times of the major lineages of the Ascomycota, because there are other reliable fossils that can be used [14,15]. However, the divergence times of the parmelioid clades may be younger than previously estimated [13]. Considering the uncertain assignment of the Miocene *Parmelia* and our new Paleogene fossils, the only reliable fossil Parmeliaceae is *Anzia*. However, the precise position of the genus inside the family is still uncertain, despite the recent phylogenetic studies.

## Conclusions

The Baltic amber inclusion described as *Alectoria succinii* Mägdefrau 1957 does not possess any bona fide anatomical features of lichens, and it probably represents a poorly preserved plant part. Hence, the fossil does not prove the presence of the genus *Alectoria* in the Paleogene. However, new fossils from Baltic and Bitterfeld amber show that lichens morphologically similar to *Alectoria* were in fact present in the Paleogene, some 24 to 35 million years ago. These new fossils either represent members of the extant genus *Oropogon* or a lineage within the alectorioid group.

The previous treatment of *Alectoria succini* points to the importance of following careful procedures for using fossils as minimum age constraints in molecular phylogenetic studies [19]. Whenever possible, the original fossil material should be re-investigated, an apomorphy-based diagnosis of the specimens should be performed in order to justify the phylogenetic position of the fossil, and the most recent geologic data should be considered.
